# The long noncoding RNA MALAT1 modulates adipose loss in cancer-associated cachexia by suppressing adipogenesis through PPAR-γ

**DOI:** 10.1186/s12986-021-00557-0

**Published:** 2021-03-10

**Authors:** Jun Han, Lei Shen, Zheng Zhan, Yuguo Liu, Chang Zhang, Ruochen Guo, Yangjun Luo, Zhiqin Xie, Ying Feng, Guohao Wu

**Affiliations:** 1 Department of General Surgery, Zhongshan Hospital, Fudan University, Shanghai, 200032 China; 2 CAS Key Laboratory of Nutrition, Metabolism and Food Safety, Shanghai Institute of Nutrition and Health, University of Chinese Academy of Sciences, Chinese Academy of Sciences, Shanghai, 200032 China

**Keywords:** Cancer-associated cachexia, Adipose loss, MALAT1

## Abstract

**Background:**

Cancer-associated cachexia is a multifactorial syndrome defined by progressive weight loss with ongoing loss of adipose tissue and skeletal muscle. Adipose loss occurs in the early stage of cachexia and is associated with reduced quality of life and survival time. Although numerous lncRNAs are regarded as novel regulators in adipose metabolism, the role of lncRNAs that selectively modulate the development of adipose loss in cachexia remains limited.

**Methods:**

In this study, we analyzed microarray data of lncRNAs in adipose loss and further explored the function and mechanism of MALAT1 in adipose loss. First, we explored the expression and function of MALAT1 in adipose cell by quantitative PCR and RNA knockdown. Subsequently, the mechanism of MALAT1 involvement in adipose loss was analyzed via RNA-seq, bioinformatics analysis and reporter gene assay. Finally, we explored the clinical significance of MALAT1 through correlation analysis.

**Results:**

Cellular experiments revealed that knocking down MALAT1 significantly inhibited the process of adipogenesis. RNA-seq data showed that numerous adipogenic genes were downregulated upon MALAT1 knockdown. A protein–protein interaction network analysis identified PPAR-γ as the central node transcription factor, the inhibition of which explains the downregulation of numerous adipogenic genes. A reporter gene assay suggested that MALAT1 can regulate the gene expression of PPAR-γ at the transcriptional level. Moreover, MALAT1 was weakly expressed in the subcutaneous white adipose tissue of cancer-associated cachexia patients and was related to low fat mass index and poor prognosis in cancer patients.

**Conclusions:**

This study indicated that MALAT1 is associated with adipose loss in cancer-associated cachexia by regulating adipogenesis through PPAR-γ, which may potentially be a novel target for the diagnosis and treatment of cancer-associated cachexia in the clinic.

**Supplementary Information:**

The online version contains supplementary material available at 10.1186/s12986-021-00557-0.

## Introduction

Cancer-associated cachexia (CAC) is a fatal energy-wasting syndrome that results in specific loss of adipose tissue and skeletal muscle [1]. Approximately 50–80% of cancer patients suffer from CAC, and 20% of cancer deaths are due to CAC [2]. CAC significantly increases therapy-related toxicity and complications but also decreases quality of life and survival time [3]. However, due to the lack of predictive biomarkers, patients are not diagnosed until their CAC symptoms have become severe. Currently, there is no specific medical treatment for CAC [1, 4]. Thus, it is vital to discover the potential mechanisms underlying CAC, which may provide a basis to develop novel diagnostic biomarkers and treatments for this devastating syndrome.

Adipose tissue plays an important role in tumors. Too much (obesity) or too little (cachexia) adipose tissue is detrimental to tumor patients. Obesity is associated with increased risk, poor prognosis and poor outcome of therapy in various cancers. Obesity-associated factors or adipokines, especially leptin and resistin, are purported to promote the growth, survival, proliferation, and invasiveness of cancer cells [5]. A study found that free fatty acids significantly increased radiation-induced cytotoxicity in cervical cancer cell lines [6]. For CAC patients, adipose tissue loss is more pronounced than muscle wasting and occurs earlier [7]. Adipose tissue loss is also associated with reduced quality of life survival time [8, 9]. Impaired adipogenesis, increased lipolysis, and browning of white adipose tissue (WAT) are the main mechanisms underlying adipose loss in CAC [10]. For example, the expression of adipogenic transcription factors such as PPAR-γ and C/EBP-α were reduced in the WAT of mice with CAC [11]. In addition, chronic inflammation mediated by interleukin-6 (IL-6) and tumor necrosis factor-alpha (TNF-α) are also an important regulators of adipose tissue loss in CAC [12–14]. Hence, identifying potential regulators in adipose tissue loss in CAC is a golden opportunity to discover ground-breaking therapeutic agents for CAC patients.

Noncoding RNAs (miRNAs, lncRNAs, etc.) have a variety of biological functions and play important roles in many diseases. For example, miR-203 is highly upregulated in breast cancer tissues and in ER-positive breast cancer cell lines. Anti-miR-203 suppresses ER-positive breast cancer growth and stemness by targeting SOCS3 [15]. LncRNAs are an emerging as novel regulators in the adipose tissue loss in CAC [16–18]. In a previous study using microarray analyses, we identified a set of differentially expressed lncRNAs in the WAT of CAC mice, compared to control mice, which included MALAT1 [17]. MALAT1 is a highly expressed lncRNA in many tissues and participates in a series of physiological and pathological processes, including myogenesis, cancer, aortic aneurysm, etc. [19–22]. For instance, the MALAT1-HuR ribonucleoprotein complex can depress CD133 expression and suppress epithelial–mesenchymal transition in breast cancer [23]. MALAT1 can regulate myogenic differentiation and muscle regeneration by modulating MyoD transcription [20]. Despite that, sequencing data showed that there was no difference in the expression of MALAT1 in the muscles of the CAC mice and control mice [24]. Although numerous lncRNAs are regarded as novel regulators in adipose metabolism, such as in WAT browning and adipogenesis [25–27], the potential role of MALAT1 in adipose tissue loss in CAC remains ambiguous.

In our current study, we explored the expression and function of MALAT1 in adipocytes. We performed knockdown experiments and determined that MALAT1 is located primarily in the nucleus and that it can suppress adipogenesis. Then, we used RNA-seq analysis to examine the levels of adipogenic genes in MALAT1-knockdown adipocytes. We found that MALAT1 can regulate the transcription of PPAR-γ, which was an upstream regulator responsible for the suppression of adipogenic genes in MALAT1 knockdown. Moreover, we observed that MALAT1 was weakly expressed in the subcutaneous WAT of CAC patients and was associated with poor prognosis in cancer patients. Thus, our work indicated that the expression of MALAT1 was associated with CAC, and that MALAT1 could be a potential target to the diagnosis and treatment of CAC.

## Methods

### Cell culture

C3H/10T1/2 (C3H10) cells were cultured in high-glucose Dulbecco’s modified Eagle medium supplemented with 10% fetal bovine serum. All cells were kept in an atmosphere of 5% CO2 and 95% air at 37 °C. Differentiation of C3H10 cells was initiated by an induction medium (0.5 mM isobutyl-1-methylxanthine, 5 mM dexamethasone, 0.5 mg/L troglitazone, 10 ng/mL insulin). Cells were then switched to maintenance medium (10 ng/mL insulin) after 2 days for further differentiation.

### Patients and sample collection

Written informed consents were obtained from all patients whose samples were used in this study, which was also approved by the Ethics Committee of Zhongshan Hospital approved this study (No. B2020-119). From January 1, 2015 to December 31, 2018, patients with colorectal cancer who underwent surgery in Zhongshan Hospital of Fudan University were included in this study. The exclusion criteria were as follows: (1) patients aged < 18 years old; (2) patients who have received preoperative radiotherapy, chemotherapy, biological therapy or emergency surgery; (3) patients with acute and chronic infection, burns and other wasting diseases; (4) patients unable to eat by mouth; (5) patients combined with other malignant tumors; (6) patients with incomplete data. According to the international consensus on CAC proposed in 2011 [28], patients included in this study were divided into two groups: non-CAC patients with weight loss ≤ 5% in recent 6 months and CAC patients with weight loss > 5% in recent 6 months. 30 patients were enrolled in each group. The clinical characteristics of patients, including weight loss (%), fat mass index and overall survival were recorded.

### RNA analyses

Total RNAs were prepared using Trizol (Thermo Fisher Scientific) and cDNA was prepared by reverse transcription according to the manufacturer’s instructions (Promega Corporation, Madison, WI). qPCR was performed using the Applied Biosystems 7900HT instruments. Relative gene expression levels were calculated using 2^−ΔΔCt^ and compared with RPL23 as internal control.

### Protein analyses

Protein lysates were prepared by RIPA Lysis Buffer (Beyotime, China). Proteins were separated by 10% SDS–polyacrylamide gel electrophoresis and transferred to a nitrocellulose membrane. Western blotting was performed using antibodies specific to the target genes. Antibody lists were shown in the previous study [17]. Tubulin expression was used as an endogenous control.

### RNA knockdown

Antisense oligonucleotides (ASOs) were ordered from RiboBio (Guangzhou, China). C3H10 cells at 70% confluence were transfected with 100 nM ASOs using RNAiMAX Transfection Reagent (Invitrogen, Carlsbad, CA, USA) according to instructions. Eight hours post-transfection, cells were recovered in full culture medium, grown to confluence, and induced to differentiation as described above.

### Reporter gene assays

The − 2 kb PPAR-γ reporter gene was amplified and cloned into pGL3 basic vector. PPAR-γ-pGL3 vector and pGL3 basic vector were transiently transfected with MALAT1 (a gift from Huating Wang [20]) or control plasmids using Lipofectamine2000 (Invitrogen) respectively. Forty-eight hours after transfection, the cells were harvested and luciferase activity was measured using the Dual-luciferase Reporter Assay System (Promega). Firefly luciferase reporter gene measurements were normalized using Renilla luciferase activity.

### Oil Red O staining

C3H10 cells were fixed with 10% formaldehyde for 30 min, washed twice with PBS, and then stained with 0.3% Oil Red O (ORO) solution. To assess lipid accumulation, the ORO retained in cells was dissolved in isopropanol and the absorbance of solution was examined at 520 nm.

### Statistics

Data were presented as means ± standard error of the mean (SEM). Statistical analyses were performed using a two-tailed Student’s t-test. The correlations of MALAT1 with weight loss and FMI were analyzed using GraphPad Prism 7 after figures were generated. Survival curves were plotted using the Kaplan–Meier method, and any difference was analyzed by the log-rank test. Statistical significance was defined as *p* < 0.05.

## Results

### The expression of MALAT1 in adipocyte

**Fig. 1:**
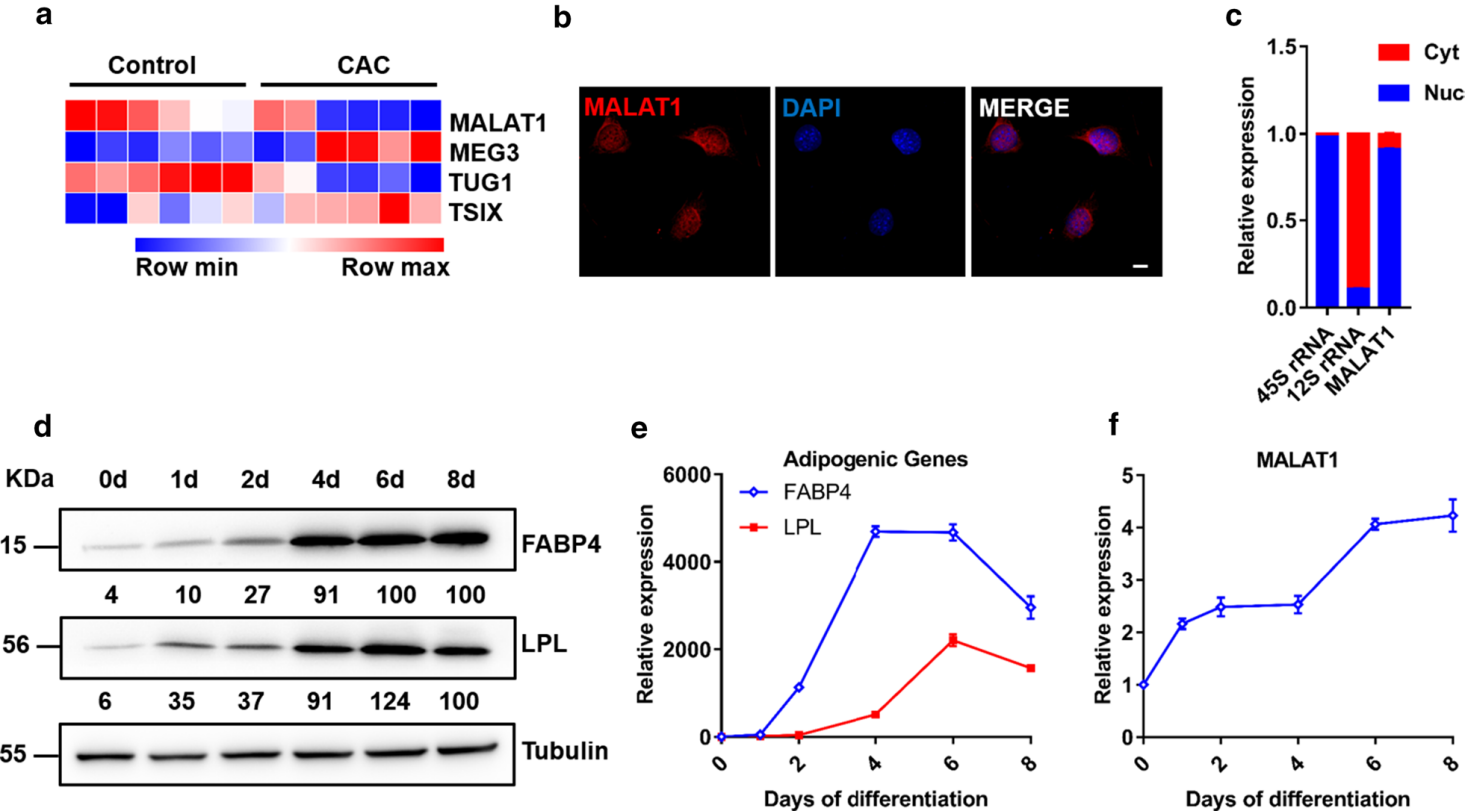
The expression of MALAT1 in adipocyte. **a** Heatmap of four lncRNAs differentially expressed in subcutaneous WAT between CAC mice and control mice. The color intensity represents the normalized intensity (n = 6). **b** RNA FISH of MALAT1 (red) in C3H10 cells. DAPI was used as counterstaining (scale bar, 10 μm). **c** qPCR analyses of nuclear (Nuc) and cytoplasmic (Cyt) fractions of differentiated C3H10 cells. 12S rRNA served as a positive control in Cyt; 45S rRNA served as a positive control in Nuc. **d**, **e** Expression of adipogenic marker genes during adipogenesis of C3H10 cells as assessed by qPCR and immunoblotting. **f** Expression of MALAT1 during adipogenesis of C3H10 cells as assessed by qPCR. The experiments were repeated 3 times. The data represent means ± SEM. n = 3

To identify lncRNAs involved in adipose tissue loss in CAC, we established a CAC mouse model using C26 colon tumor cells. Then, we performed transcriptional profiling of adipose tissues in CAC mice and control mice using microarrays that covered 35,923 annotated lncRNAs (as previously described) [17]. The data have been deposited in the Gene Expression Omnibus database under accession number GSE118611. A set of differentially expressed lncRNAs was identified, including MALAT1, MEG3, TUG1, and TSIX, which have been studied in other fields (Fig. 1a). Among subcutaneous WAT samples from CAC patients and non-CAC patients, we observed that MALAT1 was significantly downregulated in the CAC patient samples, while the other three lncRNAs either displayed much lower expression than the reference gene RPL23, or had no differential expression in the subcutaneous WAT of patients. Therefore, we focused on MALAT1 for further research. First, we analyzed the localization of MALAT1 in C3H10 cells by using RNA fluorescence in situ hybridization (FISH). In line with previous reports, MALAT1 was found exclusively in the nucleus (Fig. 1b), which was confirmed by cellular fractionation assays and qPCR analyses. As a control, 45S ribosomal RNA (rRNA) was found mainly in the nucleus (98%), whereas 12S rRNA was highly enriched in the cytoplasm (90%) (Fig. 1c). Then, we examined adipocyte gene expression at different time points following adipogenic induction. Gene expression analysis indicated that the expression of MALAT1 was significantly increased during the adipogenesis of C3H10 cells along with adipogenic marker genes, FABP4 and LPL, suggesting that MALAT1 may act as an adipogenic factor during adipogenesis (Fig. 1d–f).

### MALAT1 is required for adipogenesis

**Fig. 2:**
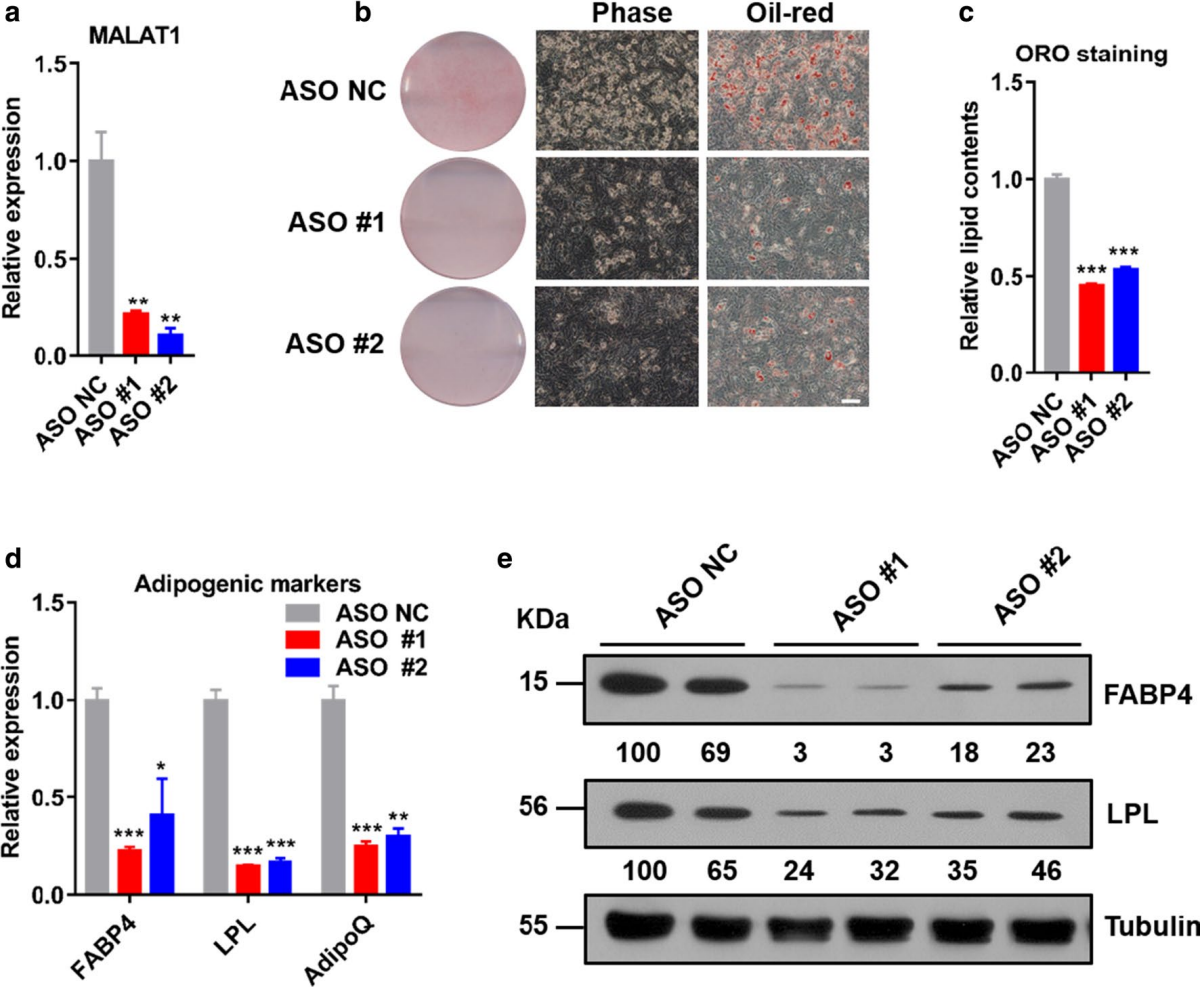
MALAT1 is required for adipogenesis. **a** Expression of MALAT1 in C3H10 cells transfected with ASO targeting MALAT1 (#1 and #2) or negative control (NC) and collected for qPCR analysis at differentiation day 6. **b** Representative images of ASO-treated C3H10 cells at differentiation day 6 labeled with Oil Red O (ORO) (scale bar, 100 μm). **c** Quantitative analysis of lipid accumulation in C3H10 cells from **b** at differentiation day 6. **d** qPCR analyses of the expression of adipogenic markers in ASO-treated C3H10 cells at differentiation day 6. **e** Immunoblot analyses of the expression of adipogenic markers in ASO-treated C3H10 cells at differentiation day 6. The experiments were repeated 3 times. The data are expressed as the means ± SEM. n = 3. *p ≤ 0.05; **p ≤ 0.01; ***p ≤ 0.001

We proceed to perform RNAi knockdown studies to assess the role of MALAT1 in adipogenesis. We designed two independent ASOs targeting MALAT1 and transfected them into C3H10 cells, followed by inducing differentiation. Approximately 80% knockdown was achieved by individual ASOs (#1, #2) at differentiation day 6 compared with the negative control (NC) (Fig. 2a). MALAT1 knockdown inhibited lipid accumulation and adipogenesis of C3H10 cells on day 6, as indicated by Oil Red O staining (Fig. 2b, c). The results were also corroborated by the decreased gene expression of adipogenic markers FABP4, LPL and AdipoQ at the RNA level (Fig. 2d). Furthermore, Western blotting confirmed decreased amounts of FABP4 and LPL proteins (Fig. 2e). In summary, loss-of-function studies illustrated that MALAT1 is required for adipogenesis in cultured cells, leading us to hypothesize that low levels of MALAT1 might play important roles in CAC-related adipose tissue loss.

### Analysis of global gene expression upon MALAT1 depletion

**Fig. 3:**
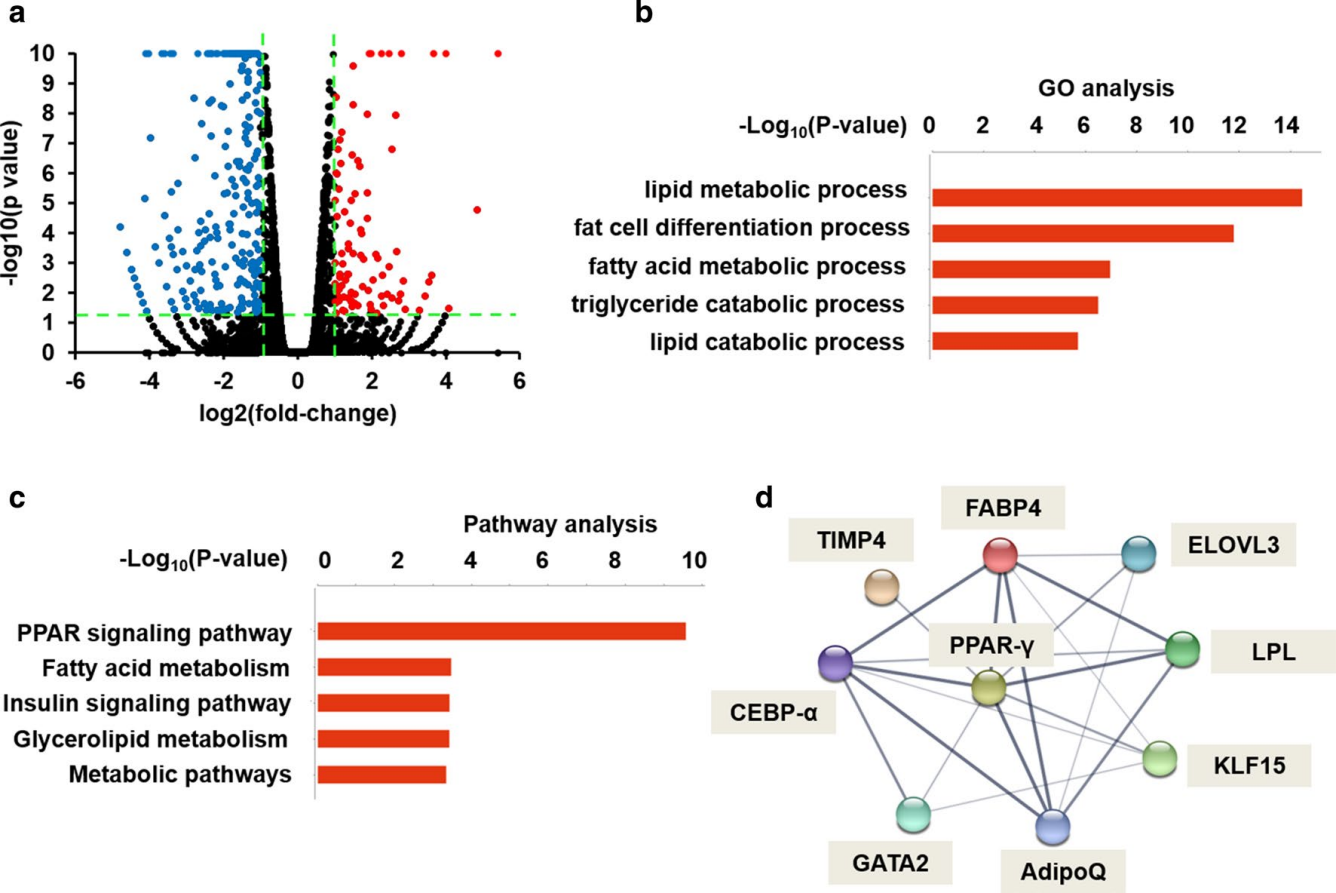
Analysis of global gene expression upon MALAT1 depletion. **a** Volcano plots showing differentially expressed genes in ASO-treated C3H10 cells at differentiation day 6. **b** Top 5 GO biological process terms changed among mRNA genes upon MALAT1 knockdown relative to the control. **c** Top 5 pathways affected by MALAT1 knockdown relative to the control. **d** The correlation of PPAR-γ and downstream adipogenic genes was demonstrated by PPI network analysis

To gain further insights into MALAT1 function, we performed RNA-seq analysis using RNA extracted from ASO-treated C3H10 cells and control cells. We identified 1120 differentially expressed genes (*p* < 0.05), comprising of 403 enriched and 717 depleted genes in MALAT1-knockdown cells, compared to control cells (Fig. 3a). Gene ontology (GO) analysis indicated that gene sets affected by MALAT1 knockdown were enriched in several biological processes, including lipid metabolism, fat cell differentiation, and fatty acid metabolism (Fig. 3b). A pathway analysis showed that the top pathways associated with MALAT1 knockdown were the PPAR signaling pathway, fatty acid metabolism, and insulin signaling pathway (Fig. 3c). The GO and pathway analyses results together revealed that the functions of genes downstream of MALAT1 are associated with adipogenesis. We thus sought to computationally identify upstream regulators that may be critical for the suppression of adipogenic genes. A protein–protein interaction (PPI) network analysis led to the identification of PPAR-γ as the central node transcription factor, the inhibition of which explaining the downregulation of numerous adipogenic genes (Fig. 3d). These results indicate that MALAT1 is a key genetic component to carry out adipogenesis.

### MALAT1 is involved in adipogenesis by regulating the transcription of PPAR-γ

**Fig. 4:**
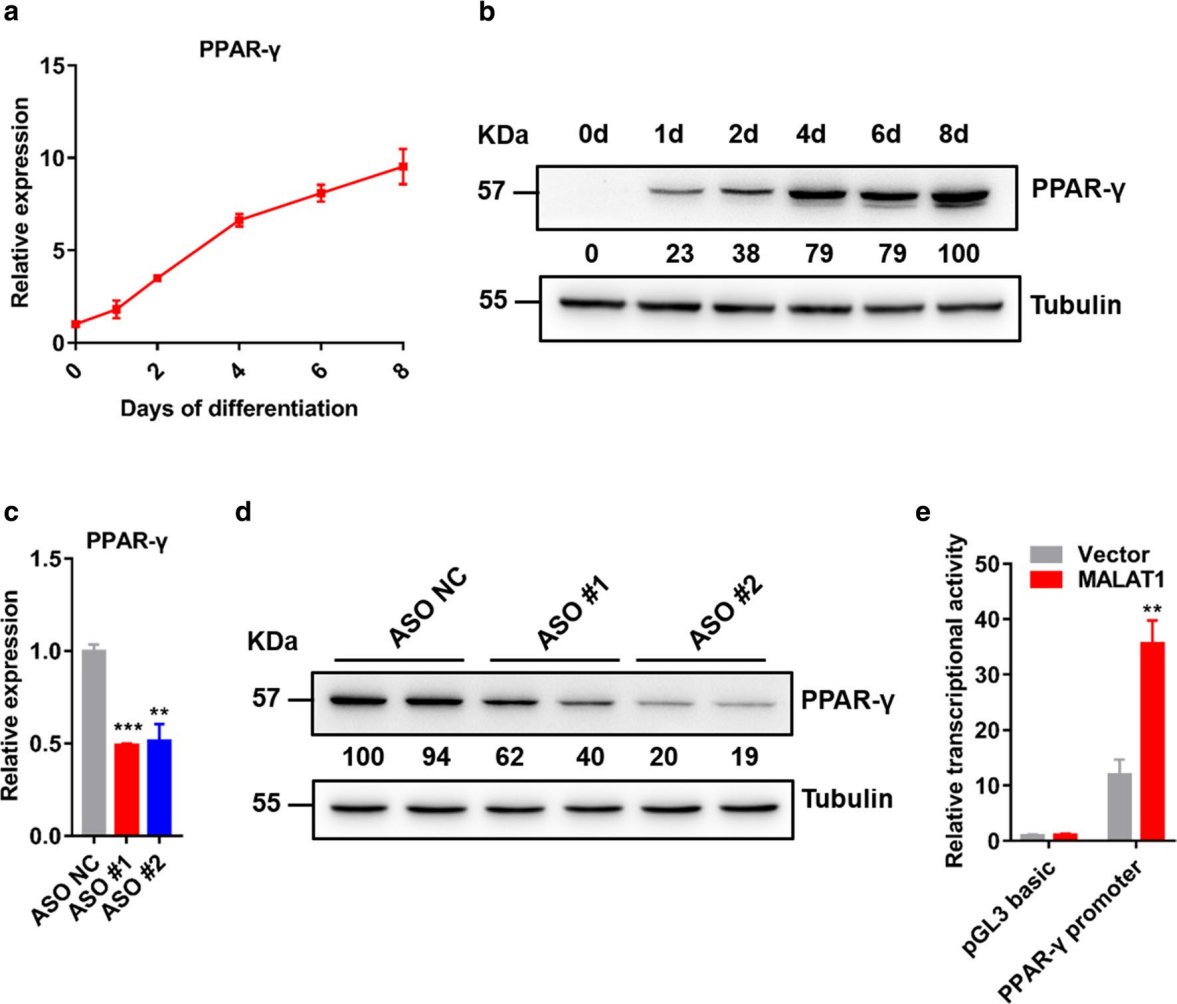
MALAT1 is involved in adipogenesis by regulating the transcription of PPAR-γ. **a** qPCR analyses of the expression of PPAR-γ during the adipogenesis of C3H10 cells. **b** Immunoblot analyses of the expression of PPAR-γ during C3H10 cells adipogenesis. **c** qPCR analyses of the expression of PPAR-γ in C3H10 cells without/with knockdown of MALAT1 at differentiation day 6. **d** Immunoblotting analyses of the expression of PPAR-γ in C3H10 cells without/with knockdown of MALAT1 at differentiation day 6. **e** The transcriptional activity of the -2 kb region of PPAR-γ in response to MALAT1 or control vector in 293 T cells. The experiments were repeated 3 times. The data represent the means ± SEM. n = 3. *p ≤ 0.05; **p ≤ 0.01; ***p ≤ 0.001

PPAR-γ is the master transcription factor of adipogenesis, leading us to suspect that the function of MALAT1 may be associated with PPAR-γ regulation. To further explore the correlation between MALAT1 and PPAR-γ, we first monitored the expression of PPAR-γ at different time points following adipogenic induction. Gene expression analysis indicated that the expression of PPAR-γ was significantly increased during adipogenesis of C3H10 cells (Fig. 4a, b). The expression pattern of PPAR-γ during adipogenesis was consistent with the expression pattern of MALAT1, as shown in Fig. 1f. Furthermore, we found that knocking down MALAT1 downregulated the mRNA and protein levels of PPAR-γ (Fig. 4c, d). To further test MALAT1 regulation of PPAR-γ transcription, 293 T cells were cotransfected with the PPAR-γ luciferase reporter in the presence of the MALAT1 vector. The MALAT1 vector significantly increased the reporter activity compared with the control vector (Fig. 4e). PPAR-γ has been reported to be the principal adipogenic transcription factor participating in the terminal process of adipogenesis; therefore, we hypothesized that MALAT1 regulates adipogenesis through PPAR-γ.

### Expression of MALAT1 in the adipose tissue of CAC patients

**Fig. 5:**
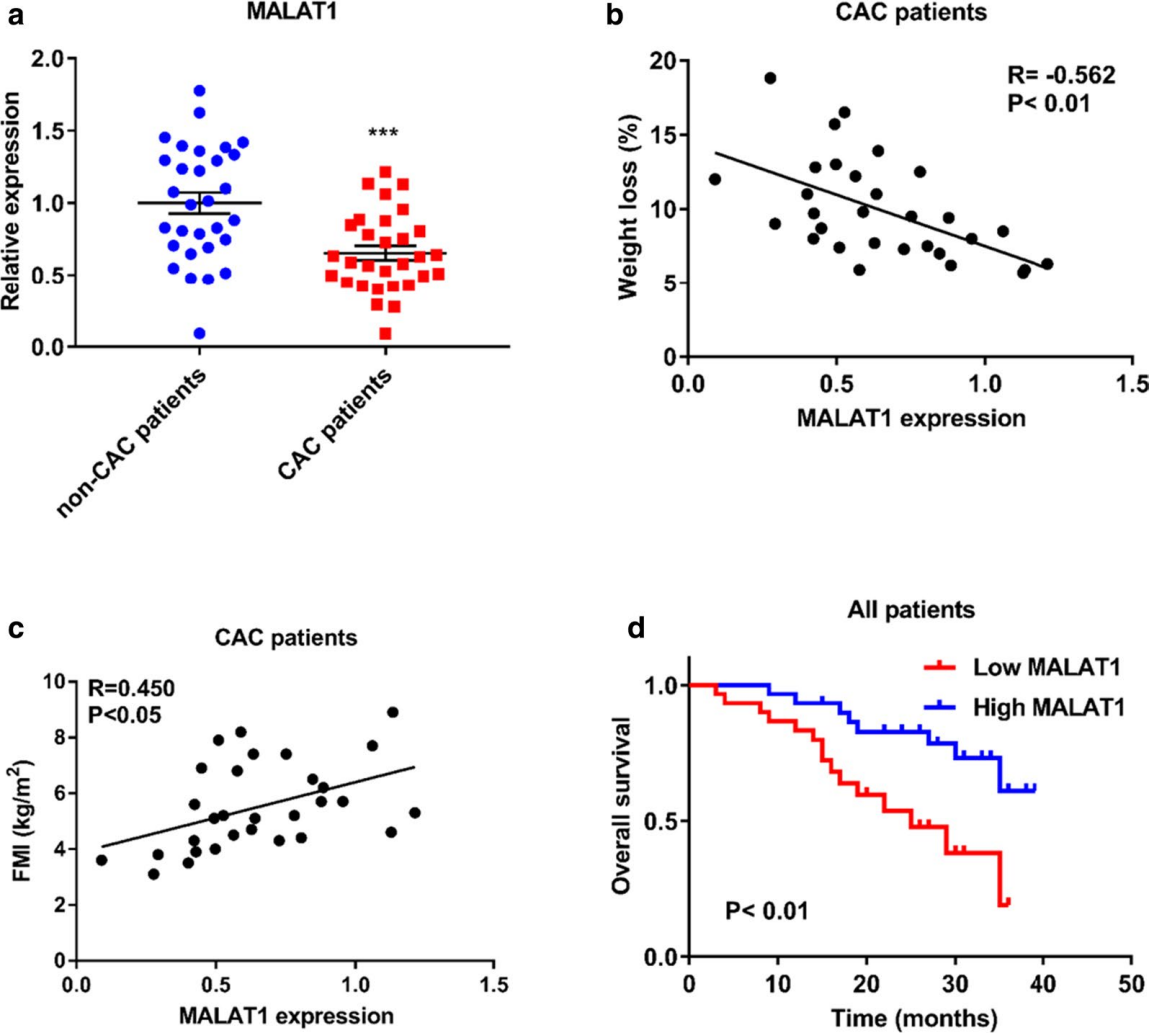
Expression of MALAT1 in the adipose tissue of CAC patients. **a** Expression of MALAT1 in the subcutaneous WAT of CAC patients (n = 30) and non-CAC patients (n = 30). **b** The correlation between the expression levels of MALAT1 and weight loss (%) in CAC patients (n = 30). **c** The correlation between the expression levels of MALAT1 and FMI in CAC patients (n = 30). **d** Kaplan–Meier survival analysis of patients with low or high expression levels of MALAT1 in adipose tissue (n = 60). The experiments were repeated 3 times. The data represent the means ± SEM. ***p ≤ 0.001

To explore the significance of MALAT1 in CAC patients, we examined the expression levels of MALAT1 in the adipose tissue of CAC patients and non-CAC patients the (patient baseline characteristics are shown in Additional file 1: Table S1). Compared with its expression in non-CAC patients, MALAT1 was weakly expressed in adipose tissues of CAC patients (Fig. 5a). Additionally, a negative correlation was observed between MALAT1 expression levels and weight loss (%) in CAC patients (Fig. 5b). Moreover, the decreased expression of MALAT1 in adipose tissue was associated with a lower fat mass index (FMI) of CAC patients (Fig. 5c). An overall survival analysis was performed via the Kaplan–Meier method, which revealed patients with low expression levels of MALAT1 in adipose tissue generally had significantly shorter overall survival times, compared to patients with high MALAT1 expression (Fig. 5d).

## Discussion

MALAT1 was originally identified due to its multiple functions in many physiological and pathological processes [20, 21, 23, 29]. The sequence of MALAT1 is highly conserved among mammals. Multiple studies have demonstrated that the inhibition of MALAT1 impairs the proliferative and invasive properties of cancer cells, which further supports the involvement of MALAT1 in cellular transformation and/or tumorigenesis [23, 30]. A recent study found that MALAT1 can regulate myogenesis by modulating MyoD transcriptional activity [20]. Though some research has reported that MALAT1-KO mice do not exhibit an obvious developmental phenotype [31], there is a hypothesis that MALAT1 displays no phenotype in living mice maintained under normal conditions; its function is thought to becomes apparent only in specific cell types or under certain pathological conditions. In this study, we found that knocking down MALAT1 significantly suppressed adipogenesis. This result cannot have been caused by off-target effects, due to the use of two independent ASOs. As MALAT1 had not been previously studied in CAC, this study pioneers the involvement of MALAT1 in adipose tissue loss in CAC by regulating adipogenesis. This discovery adds a novel function to MALAT1 as another example of lncRNA involved in adipose tissue loss in CAC.

Emerging evidence suggests that MALAT1 can regulate gene expression at the transcriptional level. For example, MALAT1 interacts with TEAD proteins and inhibits the transcription of the target genes ITGB4 and VEGFA [19]. Moreover, MALAT1 plays a critical role in pre-mRNAs metabolism. MALAT1 interacts with serine/arginine (SR) splicing factors and modulates their distribution to regulate alternative splicing of pre-mRNAs [32, 33]. In this study, we provided new insight into the molecular mechanism of MALAT1 in adipogenesis. We found that MALAT1 regulates adipogenesis by controlling the transcription of PPAR-γ, though the mechanisms by which MALAT1 regulates the transcription of PPAR-γ needs to be further studied. Previous research found that C/EBP-β can regulate the transcription of PPAR-γ, but whether MALAT1 can regulate the transcription of PPAR-γ through C/EBP-β needs to be further explored [34]. In addition, inflammatory cytokines such as interleukin-6 and tumor necrosis factor-α are also associated with adipose tissue loss in CAC [35]. With regards to whether these cytokines can influence the expression of MALAT1 and thus regulate adipogenesis calls for further research.

Various proteins are known to participate in adipose tissue loss in CAC, including HSL, ATGL, UCP1 [36–38]. In addition to the aforementioned proteins, lncRNAs such as CAAlnc1 are emerging as novel regulators in the adipose loss in CAC [17]. In our study, we found that MALAT1 was weakly expressed in the subcutaneous WAT of CAC patients and was closely associated with low FMI and poor prognosis in cancer patients. Therefore, the expression level of MALAT1 is an important prognostic factor for tumor patients. Some preclinical studies are focusing on the treatment of CAC. For example, Ampk inactivation was correlated with the adipose tissue loss in CAC. An AMP-activated protein kinase–stabilizing peptide was able to ameliorate WAT wasting in vitro and in vivo by shielding the Cidea-targeted interaction surface on Ampk [39]. For MALAT1, much work remains to be done in order to confirm its value in the adipose tissue loss in CAC.

## Conclusions

This study revealed that MALAT1 was weakly expressed in the subcutaneous WAT in CAC patients and its association with a low index of fat mass and poor prognosis in cancer patients. The knockdown of MALAT1 was observed to suppress adipogenesis by regulating the expression of PPAR-γ at the transcriptional level. Therefore, this study provides a potential target for the diagnosis and treatment of patients with CAC.

## Supplementary Information

**Additional file 1: Table S1.** Patient baseline characteristics.

## Data Availability

RNA-seq data are deposited at the Gene Expression Omnibus database with the accession number GSE156615. All data generated and analysed during the current study are available from the corresponding author on reasonable request.

## Abbreviations

CAC: Cancer-associated cachexia
WAT: White adipose tissue
IL-6: Interleukin-6
TNF-α: Tumor necrosis factor-alpha
SR: Serine/arginine
GO: Gene ontology
NC: Negative control
rRNA: Ribosomal RNA
ASO: Antisense oligonucleotide
FISH: Fluorescence in situ hybridization
SEM: Standard error of the mean
PPI: Protein–protein interaction
Nuc: Nuclear
Cyt: Cytoplasmic
ORO: Oil-Red O
