# Supplementary material for: The long noncoding RNA MALAT1 modulates adipose loss in cancer-associated cachexia by suppressing adipogenesis through PPAR-γ
Source: Nutr Metab (Lond). 2021 Mar 10;18:27. doi: 10.1186/s12986-021-00557-0 (PMC7944636; doi:10.1186/s12986-021-00557-0)
Supplement: Supplementary file 1 — Additional file 1: Table S1. Patient baseline characteristics. [file 12986_2021_557_MOESM1_ESM.pdf]

Table S1. Patient baseline characteristics

Clinical Characteristics	Non-CAC patients (n=30) No (%)	CAC patients (n=30) No (%)	Test of significance
**Gender**			
Male	19 (63.3%)	21 (70.0%)	p=0.5839
Female	11 (36.7%)	9 (30.0%)	
**Age**			
≤60	17(56.7%)	20(66.7%)	p=0.4257
＞60	13(43.3%)	10(33.3%)	
**Tumor site**			
Colon	15(50.0%)	13(43.3%)	p=0.6048
Rectum	15(50.0%)	17(56.7%)	
**T stage**			
T1-2	—	—	p=0.7656
T3	8(26.7%)	7(23.3%)	
T4	22(73.3%)	23(76.7%)	
**N stage**			
N0	13(43.3%)	9(30.0%)	p=0.2945
N1	10(33.3%)	16(53.3%)	
N2	7(23.3%)	5(16.7%)	
**M stage**			
M0	19(63.3%)	14(46.7%)	p=0.1944
M1	11(36.7%)	16(53.3%)	
